# Cannabidiol Exposure During the Mouse Adolescent Period Is Without Harmful Behavioral Effects on Locomotor Activity, Anxiety, and Spatial Memory

**DOI:** 10.3389/fnbeh.2021.711639

**Published:** 2021-08-26

**Authors:** J. S. Kaplan, J. K. Wagner, K. Reid, F. McGuinness, S. Arvila, M. Brooks, H. Stevenson, J. Jones, B. Risch, T. McGillis, R. Budinich, E. Gambell, B. Predovich

**Affiliations:** ^1^Department of Psychology, Behavioral Neuroscience Program, Western Washington University, Bellingham, WA, United States; ^2^Department of Psychology, Experimental Psychology Graduate Program, Western Washington University, Bellingham, WA, United States; ^3^Prelabs, Lehigh Acres, FL, United States

**Keywords:** cannabidiol, anxiety, cannabinoid, cannabis, development, adolescence, learning, weight

## Abstract

Cannabidiol (CBD) is a non-intoxicating phytocannabinoid whose purported therapeutic benefits and impression of a high safety profile has promoted its increasing popularity. CBD’s popularity is also increasing among children and adolescents who are being administered CBD, off label, for the treatment of numerous symptoms associated with autism spectrum disorder, attention deficit hyperactivity disorder, anxiety, and depression. The relative recency of its use in the adolescent population has precluded investigation of its impact on the developing brain and the potential consequences that may present in adulthood. Therefore, there’s an urgency to identify whether prolonged adolescent CBD exposure has substantive impacts on the developing brain that impact behavioral and cognitive processes in adulthood. Here, we tested the effect of twice-daily intraperitoneal administrations of CBD (20 mg/kg) in male and female C57BL/6J mice during the adolescent period of 25–45 days on weight gain, and assays for locomotor behavior, anxiety, and spatial memory. Prolonged adolescent CBD exposure had no detrimental effects on locomotor activity in the open field, anxiety behavior on the elevated plus maze, or spatial memory in the Barnes Maze compared to vehicle-treated mice. Interestingly, CBD-treated mice had a faster rate of learning in the Barnes Maze. However, CBD-treated females had reduced weight gain during the exposure period. We conclude that prolonged adolescent CBD exposure in mice does not have substantive negative impacts on a range of behaviors in adulthood, may improve the rate of learning under certain conditions, and impacts weight gain in a sex-specific manner.

## Introduction

Cannabidiol (CBD) is one of the most abundant cannabinoids naturally produced by the plant, *Cannabis sativa*, and the dominant phytocannabinoid produced by the hemp variety (Citti et al., 2019). CBD’s increase in popularity over the last decade (Leas et al., 2019) is driven by easier access to CBD-containing products (McGregor et al., 2020) and claims of therapeutic efficacy (New data show Americans are turning to CBD as a cure-all for the modern condition - The Harris Poll; Sholler et al., 2020) coupled with a purported belief that CBD consumption is safe (Larsen and Shahinas, 2020). While adverse effects may occur at therapeutically relevant doses of CBD in adults (Huestis et al., 2019), less is known about its long-term consequences on the developing brain.

Intractable pediatric epilepsies are the only currently approved therapeutic uses of CBD, Epidolex, by the United States Food and Drug Administration. Yet, anxiety, sleep problems, and stress are the most commonly reported reasons for use (Moltke and Hindocha, 2021). The perception of CBD’s therapeutic benefits and high safety profile have led to off-label CBD administration in children for treating symptoms of numerous conditions including anxiety, hyperactivity, and autism spectrum disorder. Early-stage clinical trials highlight the potential benefits of CBD-based therapies on secondary symptoms of autism spectrum disorder such as reducing hyperactivity, the frequency of self-injury rage attacks, sleep-disturbances, and anxiety (Aran et al., 2018; Barchel et al., 2019). Somnolence is often reported as one of the more common acute centrally mediated adverse events of CBD use in children and adults (Devinsky et al., 2017; Szaflarski et al., 2018; Barchel et al., 2019), but the frequency of overall adverse effects seems to increase with dose and additional pharmacotherapies (Devinsky et al., 2017). Few studies have tracked symptoms after more than several months of CBD treatment and all have been conducted too recently to track participants into adulthood. Although one notable study found that cognitive performance was unaltered after one year of CBD treatment (Epidolex) in a cohort of children with treatment-resistant epilepsy (Thompson et al., 2020), little is known about long-term consequences that may persist or present in adulthood.

CBD also has antidepressant (Linge et al., 2016), anti-inflammatory (Mecha et al., 2013), and immunomodulator effects (Kozela et al., 2010) in experimental models of disease through mechanisms different than traditional therapeutics (Silvestro et al., 2020). CBD has over 65 known targets in the brain and body that include a variety of receptors, ion channels, enzymes, and transporters (Ibeas Bih et al., 2015). These targets are found throughout the brain and in high density in the prefrontal cortex, hippocampus, striatum, and cerebellum (Fogaça et al., 2014; Warden et al., 2016; Ashton et al., 2017; Zuardi et al., 2017; Meyer et al., 2018; Barnes et al., 2020) and commonly affect intracellular calcium signaling (Ibeas Bih et al., 2015). Therefore, we tested the hypothesis that CBD exposure during a period of heightened neuroplasticity will affect gross behaviors associated with locomotor activity, spatial learning and memory, and anxiety.

Few studies have investigated the effect of repeated CBD exposure on the developing brain in healthy mice. One study found that 6 weeks of daily i.p. 20 mg/kg CBD injections starting at 3-months in C57BL/6J mice was without adverse impact on behavioral measures of locomotor activity, spatial memory, and anxiety (Schleicher et al., 2019). Notably, this age range is just beyond what’s considered to be the equivalent adolescent developmental stage in mice (Brust et al., 2015). A second study looked earlier in the developmental window starting at 4 weeks and found that a lower dose of 3 mg/kg CBD delivered i.p. daily for 3 weeks, on its own, had no effects on similar behaviors, yet it protected against deficits caused by concurrent THC exposure (Murphy et al., 2017). Given that (1) the half-life of CBD in mice is shorter than in humans (Deiana et al., 2012; Millar et al., 2018), and (2) children exposed to CBD for therapeutic purposes would experience more persistently elevated levels throughout the day, it is important that mouse studies of developmental CBD exposure more closely model the higher levels of CBD humans are achieving.

There are currently no known studies to have investigated the effect of multiple daily CBD exposures during the important developmental period of adolescence in mice. We aimed to identify if CBD use during this developmental stage has harmful impacts on specific behaviors by more closely modeling CBD dosing used clinically, on or off-label, as a first step to identifying potential long-term consequences of CBD consumption in a human population. To achieve this, we tested the effect of twice-daily exposures of a 20 mg/kg CBD dose during the period of mouse adolescence on the behavioral expression of locomotor activity, anxiety, and spatial memory in adulthood.

## Materials and Methods

### Animals

C57BL/6J (Jackson Laboratories, Bar Harbor, ME) litters were bread in-house at Western Washington University. For developmental exposure studies, a total of 26 mice across 3 litters were used [16 males (8 vehicle treated, 8 CBD treated), 10 females (5 vehicle treated, 5 CBD treated)] in a between-subjects experimental design. All procedures conform to the regulations detailed in the National Institutes of Health *Guide for the care and use of laboratory animals* and were approved by the Institutional Animal Care and Use Committee at Western Washington University. Litters were weaned at postnatal day (PND) 21, separated by sex, and housed in groups of 3–5 mice per cage on a 12 h light/dark cycle (lights on at 0700). Food and water were provided *ad libitum*. All drug exposures and behavioral testing were conducted during the light cycle. For the acute anxiety study in adult mice, male and female mice aged PND 100–150 and matched for body weights (*P* = 0.82 by one-way ANOVA) were tested.

### Drug Administration

CBD isolate (>98% purity) was purchased from Cayman Chemical Company (Ann Arbor, MI) and dissolved in a vehicle solution of 1:1:18 ethanol:cremophor:0.9% saline at a concentration of 3 mg/ml. Mice were i.p. injected twice daily (0900 and 1700) for 21 days with either 20 mg/kg CBD or an equivalent volume of vehicle solution based on body weight measurements obtained each morning. No further drug exposure occurred after the 3-week exposure period.

### Behavioral Assessment

Behavioral assessment began at PND 60 with the open field test, followed by the elevated plus maze (PND 65), and lastly the Barnes Maze Test of Spatial Learning (PND 70–74). Behavioral assays were conducted by experimenters blind to the experimental condition. Animal movement was recorded in the presence of overhead fluorescent light using a digital camera (Microsoft LifeCam) mounted above the behavioral apparatus. Behavior was analyzed using ezTrack open source animal tracking software (Pennington et al., 2019). Each video was checked for accurate assessment by visually inspecting output bokeh plots and calculating total ratios to ensure that 100% of their behavior was captured in analysis. At the end of each trial, the behavioral apparatus was cleaned with 70% ethanol and wiped with paper towels.

### Open Field

Each mouse was placed near the same wall of the 44 × 44 cm white plexiglass open field arena and left to explore for 10 min. A center quadrant (a 22 × 22 cm square centered 11 cm from each wall) was created using the ezTrack software to measure time spent in the center of the chamber. Total distance traveled and time in the center quadrant were the primary dependent variables. Average moving speed was also assessed by limiting the analysis of locomotor activity to the center quadrant. Experimenters left the behavioral room during the experiment and monitored behavior on a computer monitor through a narrow window. The open field test was conducted with full overhead lighting.

### Elevated Plus Maze (EPM)

Subjects were placed in the center of the white plus-shaped maze and allowed to explore for 5 min. Each of the four maze arms is 60 cm × 6 cm connected in the middle at a 6 × 6 cm open center (total 126 cm in length). Two “closed” arms are surrounded by 21 cm opaque plexiglass walls on three sides while the other two “open arms” are open on all sides. The maze is elevated 93 cm above the floor. The total time spent in the open arms and the ratio of time spent in the open arms/closed arms were assessed. Experimenters left the behavioral room during the experiment and monitored behavior on a computer monitor through a narrow window. For the acute CBD assessment, mice were injected i.p. one hour prior to behavioral testing, which represents the brain *T*_max_ for i.p. CBD injections in mice (Deiana et al., 2012). In the developmental study, two mice jumped off the maze within the first couple minutes of the test and were excluded from the analysis (one from each group). The EPM test was conducted with full overhead lighting.

### Barnes Maze

The Barnes Maze is a circular planar white plexiglass platform (92 cm diameter) elevated 88 cm off the ground with 20 circular holes (7 cm diameter) spaced evenly near the perimeter. A black escape box (15 × 7 × 7 cm) was placed under one hole; its position did not change throughout the entirety of the training sessions. The entirety of the experiment lasted for five consecutive days comprised of a habituation day (day 1), three training days (days 2–4), and the probe day (day 5). On the habituation day, each mouse explored the Barnes circular maze for 3 min without the escape box present. Each of the subsequent training days were comprised of three trials each (9 total training trials) with an inter-trial interval of 20 min. At the beginning of each training trial, the subject was placed at the center of the maze and allowed to search for the target hole, beneath which was the escape box, for 3 min. If the mouse failed to find the target hole by the end of the trial, the experimenter gently guided the mouse to the hole with their hands. Once the mouse entered the escape box, the experimenter turned off the overhead lights and the mouse remained undisturbed for 1 min. During the training trials, latency, distance traveled to the target hole, and number of errors were the primary dependent measures. Mean latencies were calculated for each subject for each of the three training days. On the probe day, the escape box was removed, and each subject was allowed to look for the target hole for 90 s. For the probe trial, latency and distance traveled to find the target hole were the primary dependent variables. The Barnes Maze procedure was conducted with full overhead lighting.

### Statistical Analysis

All data are shown as mean ± s.e.m. and analyzed by either unpaired *t*-tests or ANOVA using Sigma Plot software (SPSS Inc.) with an alpha set at 0.05. When appropriate, we used two-way between subjects ANOVAs to assess the effect of sex and condition on the dependent variables. For the Barnes Maze, we used a three-way ANOVA to assess the effect of sex, exposure condition, and training day on the dependent variables with Tukey’s HSD *post hoc* comparisons to analyze main effects and interactions. Descriptive statistics (mean ± SEM) are included in the figure legends.

## Results

### Acute CBD Administration Reduces Anxiety Within a Narrow Dose Window

**FIGURE 1:**
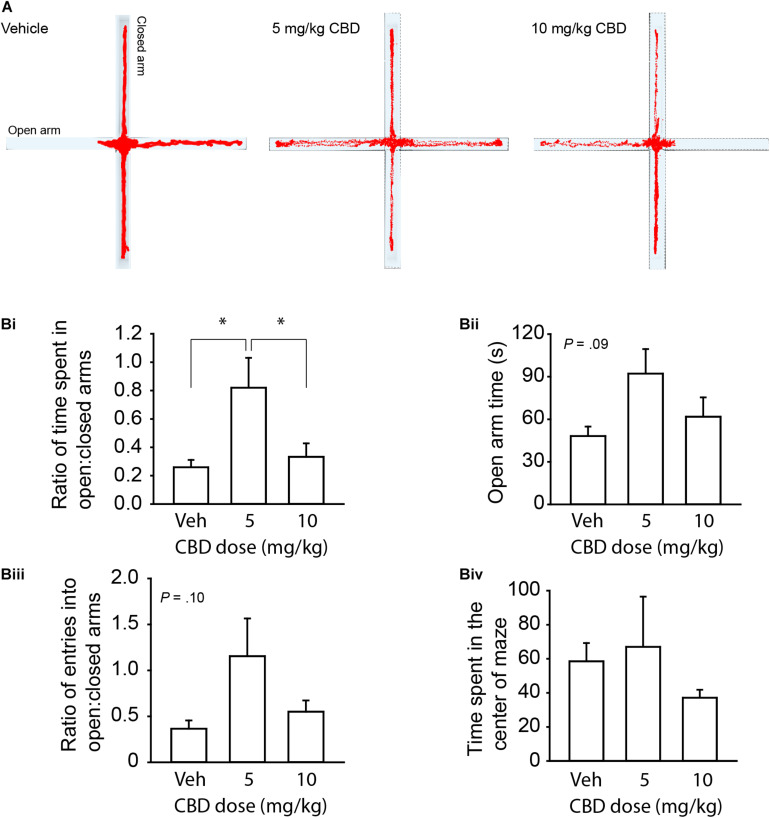
CBD dose dependently reduces anxiety-like behavior on the EPM in adult mice. **(A)** Representative activity traces of subjects administered vehicle, 5 mg/kg CBD, and 10 mg/kg CBD. **(Bi)** Summary bar chart showing that 5 mg/kg CBD increased the ratio (left) of time spent in the open arm relative to the closed arm (0.82 ± 0.21, *n* = 6) compared to vehicle (0.26 ± 0.05, *n* = 6) and 10 mg/kg (0.33 ± 0.10, *n* = 6) treated mice. **(Bii)** Summary bar chart showing the trend (*P* = 0.09) for CBD to affect time spent in the open arms by one-way ANOVA. 5 mg/kg treated mice spent more time in the open arm (92.10 ± 17.31 s) compared to vehicle (48.00 ± 6.78 s) and 10 mg/kg (61.8 ± 13.68 s) treated mice. **(Biii)** Summary bar chart showing a trend (*P* = 0.10) for CBD to affect the ratio of entries into the open:closed arms of the EPM by one-way ANOVA. 5 mg/kg treated mice had a higher ratio of entries into the open arms compared to the closed arms (1.16 ± 0.41) compared to vehicle (0.37 ± 0.09) and 10 mg/kg (0.55 ± 0.12) treated mice. **(Biv)** Summary bar chart showing that there were no differences between treatment groups in time spent in the center square of the EPM. ^∗^indicates *P* < 0.05 by Tukey’s HSD *post hoc* comparisons.

We first sought to validate the centrally mediated effects of our CBD compound from i.p. injection since previous investigation of this compound focused on topical application or *in vitro* effects (Kozela et al., 2015; Thapa et al., 2018). Because anxiety-related symptoms are common reasons for adolescent use of CBD (Masataka, 2019; Cumbo et al., 2021), we tested anxiety-related behavior in adult mice. We chose to use the EPM because of its strong reliability for assessing the behavioral effects of cannabinoids (Onaivi et al., 1990). In support of our CBD compound’s psychoactivity, a one-way between-subjects ANOVA and Tukey’s *post hoc* comparisons revealed that a 5 mg/kg dose, but not 10 mg/kg, increased the ratio between time spent in the open to closed arms relative to the vehicle, *F*(2, 15) = 4.95, *P* < 0.022 (Figures 1A,Bi). There were trending effects of CBD administration on time spent in the open arm, *F*(2,15) = 2.86, *P* = 0.09 (Figure 1Bii) and ratio of entries into the open:closed arms, *F*(2,15) = 2.67, *P* = 0.10 (Figure 1Biii). There were no differences in time spent in the center quadrant, *P* = 0.51 (Figure 1Biv). This result confirmed the psychoactive efficacy of our CBD compound and supported the investigation of a suprathreshold dose (20 mg/kg) for reducing anxiety in healthy C57BL/6J mice that is either at or above the effective dose detected in previous studies in rats (Guimarães et al., 1990; Resstel et al., 2009) and mice (Schiavon et al., 2016; Zieba et al., 2019).

### Developmental CBD Exposure Reduces Weight Gain in Female Mice Only

We next tested the effect of 3 weeks of twice daily 20 mg/kg CBD administrations, delivered i.p., during the period of mouse adolescence (PND 25–45) based on body weight measurements taken at the start and the end of the exposure period. A two-way ANOVA revealed an interaction between mouse sex and treatment group, *F*(1,22) = 6.78, *P* < 0.016, driven by reduced weight gain in the CBD-treated female mice (Supplementary Figure 1). These findings suggest that CBD may differentially impact weight gain during the adolescent period between males and females.

### Developmental CBD Exposure Does Not Impact Locomotor Activity or Anxiety

**FIGURE 2:**
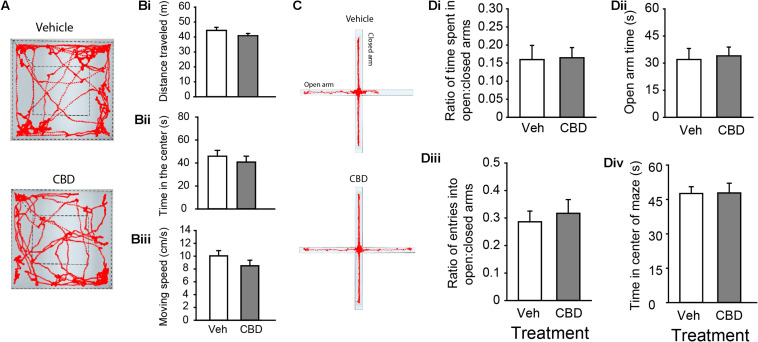
Adolescent CBD exposure does not impact locomotor or anxiety-related behaviors. **(A)** Representative activity traces in the open field of subjects administered vehicle (top) and CBD (bottom). Summary bar charts indicating that there were no difference in distance traveled between vehicle (white; 44.40 ± 2.04 m) and CBD (gray; 40.84 ± 1.53 m) treated groups **(Bi)**. There was also no difference in time spent in the center quadrant of the open field chamber between the vehicle (45.91 ± 5.10 s) and CBD (40.81 ± 5.10 s) treated groups **(Bii)**. There was no difference in the mean moving speed between vehicle (10.05 ± 0.82 cm/s) and CBD (8.51 ± 0.86) treated groups **(Biii)**. **(C)** Representative activity traces in the EPM of subjects administered vehicle (top) and CBD (bottom). Summary bar charts indicating that there was no difference in the ratio of time spent in the open arms relative to the closed arms between treatment groups (**Di**; vehicle: 0.16 ± 0.04; CBD: 0.17 ± 0.03). There was no difference in the time spent in the open arms of the EPM (**Dii**; vehicle: 32.10 ± 6.09 s; CBD: 33.9 ± 4.86 s). There was no difference in the ratio of entries into the open arms relative to the closed arms (**Diii**; vehicle: 0.29 ± 0.04; CBD: 0.32 ± 0.05), nor was there a difference in the time spent in the center square of the EPM (**Div**; vehicle: 47.63 ± 2.95 s; CBD: 47.86 ± 4.27 s).

We began our behavioral assays in adult mice starting at PND 60. There was no main effect of sex in any of our measures on the open field and EPM, all (*P* > 0.05). CBD treatment had no impact on total distance traveled (Figures 2A,Bi and Supplementary Figure 2Ai), time spent in the center quadrant of the open field (Figure 2Bii and Supplementary Figure 2Aii), nor mean moving speed through the center quadrant of the open field (Figure 2Biii and Supplementary Figure 2Aiii), all (*P* < 0.05). CBD treatment also had no effect on the ratio of time spent exploring the open arms relative to the closed arms in the EPM (Figures 2C,Di and Supplementary Figure 2Bi), time spent in the open arms (Figure 2Dii and Supplementary Figure 2Bii), the ratio of entries into the open:closed arms (Figure 2Diii and Supplementary Figure 2Biii), or the time in the center square of the EPM (Figure 2Div), all (*P* > 0.05). We conclude that prolonged adolescent CBD exposure does not negatively impact locomotor activity or anxiety-related behaviors in adulthood.

### Developmental CBD Exposure Hastens Learning of a Spatial Memory Task

**FIGURE 3:**
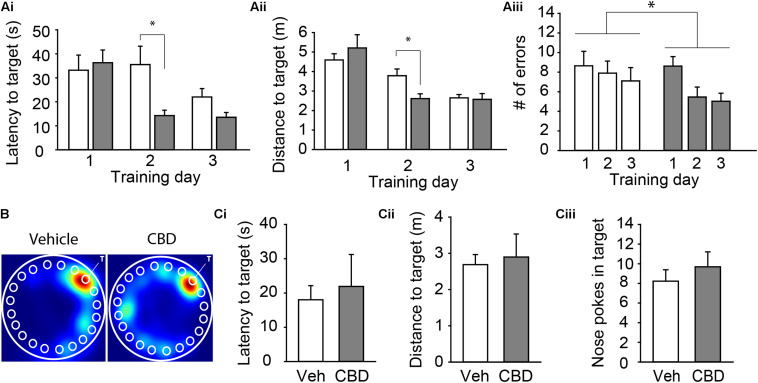
Adolescent CBD exposure improves the rate of learning of a spatial memory task. Summary bar charts showing the mean latency **(Ai)** and mean distance traveled **(Aii)** to the escape box as well as the mean number of errors **(Aiii)** on each day of acquisition. CBD-treated mice had shorter latency to target (14.29 ± 4.85 s) and distance to target (2.61 ± 0.38 m) on day 2 compared to vehicle treated mice (38.09 ± 5.05 s; 3.78 ± 0.39 m). CBD treated mice made fewer errors across all acquisition days (6.37 ± 0.59 errors) than vehicle-treated mice (7.88 ± 0.77 errors). **(B)** Representative heat maps in the Barnes maze on the probe day of subjects administered vehicle (left) and CBD (right). The white “T” indicates target hole. Summary bar charts showing that there was no difference in the latency to the target hole **(Ci)**, distance to the target hole **(Cii)**, or number of nose pokes into the target hole **(Ciii)** on the probe day between the vehicle (18.04 ± 4.12 s; 2.69 ± 0.28 m; 8.23 ± 1.16 nose pokes) and CBD (21.93 ± 0.93 s; 2.90 ± 0.64 m; 9.69 ± 1.52 nose pokes) treated groups. ^∗^indicates *P* < 0.05 by Tukey’s HSD *post hoc* comparisons.

For our last behavioral assessment, we tested spatial learning and memory on the Barnes Maze. We compared the mean latency, the distance traveled to the target hole, and the number of errors across each of the three training days to assess acquisition rate (see Table 1 for descriptive statistics). Three-way ANOVA revealed that there was a significant interaction between the exposure condition and the acquisition day for the latency to the escape box, *F*(2,66) = 5.14, *P* < 0.01 (Figure 3Ai and Supplementary Figure 3Ai), and the distance to the escape box, *F*(2,66) = 3.60, *P* = 0.04 (Figure 3Aii and Supplementary Figure 3Aii). Tukey’s *post hoc* comparisons revealed that CBD-treated mice had a shorter mean latency and mean distance to the escape box on the second training day, suggesting a faster acquisition rate of the spatial learning task, all *P* < 0.05. Further, there was a significant main effect of exposure condition on mean latency to the escape box, *F*(1,66) = 6.01, *P* = 0.02 (Figure 3Ai and Supplementary Figure 3Aii), and number of errors, *F*(1,66) = 4.04, *P* < 0.05 (Figure 3Aiii and Supplementary Figure 3Aiii). Tukey’s *post hoc* comparisons found that CBD exposed mice had a shorter mean latency to the escape box and made fewer errors. For all dependent measures, there was no main effect of sex (all *P* > 0.05; Supplementary Figure 3), but there was a significant interaction between sex and exposure condition on number of errors, *F*(1,66) = 4.04, *P* = 0.04 (Supplementary Figure 3Aiii). In this case, CBD-treated female mice made fewer errors than vehicle-treated female mice, *P* = 0.01, and there was a trend that female CBD-treated mice made fewer errors than male CBD-treated mice, *P* = 0.06. On the probe day, there was no main effect of sex, exposure condition, or an interaction between these two variables on the latency to the target hole, distance to the target hole, or the number of nose pokes into the target hole, all *P* > 0.05 (Figures 3B,C and Supplementary Figure 3B). These findings suggest that adolescent CBD exposure does not negatively impact spatial memory and may actually improve learning rates under certain conditions.

**TABLE 1:** Descriptive statistics for the Barnes Maze acquisition period.

Condition	Sex	Acquisition day	Mean latency to escape box (s)	SEM
Vehicle	M	1	25.48	7.95
Vehicle	M	2	27.47	9.17
Vehicle	M	3	18.36	4.23
CBD	M	1	42.61	7.41
CBD	M	2	17.60	2.85
CBD	M	3	16.77	2.49
Vehicle	F	1	45.48	8.52
Vehicle	F	2	48.46	12.37
Vehicle	F	3	27.93	5.42
CBD	F	1	26.42	4.53
CBD	F	2	8.61	1.15
CBD	F	3	8.56	1.86

**Condition**	**Sex**	**Acquisition day**	**Mean distance to escape box**	**SEM**

Vehicle	M	1	4.67	0.46
Vehicle	M	2	3.74	0.31
Vehicle	M	3	2.66	0.22
CBD	M	1	5.24	0.80
CBD	M	2	2.59	0.28
CBD	M	3	2.28	0.27
Vehicle	F	1	4.48	0.46
Vehicle	F	2	3.85	0.77
Vehicle	F	3	2.63	0.29
CBD	F	1	5.15	1.34
CBD	F	2	2.65	0.51
CBD	F	3	3.04	0.63

**Condition**	**Sex**	**Acquisition day**	**Mean number of errors**	**SEM**

Vehicle	M	1	7.79	2.28
Vehicle	M	2	7.58	1.18
Vehicle	M	3	6.67	1.98
CBD	M	1	9.00	1.31
CBD	M	2	6.88	1.36
CBD	M	3	6.29	1.04
Vehicle	F	1	10.00	1.33
Vehicle	F	2	8.40	2.80
Vehicle	F	3	7.80	1.80
CBD	F	1	8.00	1.56
CBD	F	2	3.20	0.87
CBD	F	3	3.00	0.78

## Discussion

The popularity of CBD is rapidly escalating (Leas et al., 2019). Epidolex, a CBD-rich cannabis extract, has been approved by the United States for the treatment of a narrow classification of pediatric epilepsies (Franco et al., 2021). However, its unapproved uses for the treatment of pain, anti-emesis, anxiety, sleep, and stress reduction has led to an increase in CBD exposure during infancy, childhood, and adolescence (Aran et al., 2018; Bertrand et al., 2018; Sarrafpour et al., 2020; Moss et al., 2021). Although many early studies point to benefits in children and adolescents across numerous indications such as epilepsy, anxiety, hyperactivity, and autism spectrum disorder (Shannon and Opila-Lehman, 2016; Aran et al., 2018; Bar-Lev Schleider et al., 2019; Barchel et al., 2019; Masataka, 2019; Poleg et al., 2019; Franco et al., 2021), CBD’s therapeutic potential could be thwarted if adverse developmental consequences are observed.

The impact of CBD on the developing brain is not well-characterized, and our understanding of its consequences has been limited, in part, by the relative recency of its popularity and the lack of data collection at long-term longitudinal time points. Here, we used a well-characterized normally developing healthy mouse model and exposed them to repeated suprathreshold anxiolytic doses of CBD for a prolonged 3-week time course during the mouse adolescent period (Brust et al., 2015). This is the first study to assess the impact of multiple daily CBD doses in mice during this important developmental stage. Although we detected less weight gain during the duration of our study in CBD-treated female mice, we failed to detect any detrimental effects on general locomotor activity, anxiety, or learning and spatial memory. Surprisingly, we revealed an improvement in the learning rate of a spatial memory task in CBD-treated mice compared to those administered the vehicle.

CBD is currently being used off-label or under investigation for treating numerous conditions in children and adolescents including autism-spectrum disorder (Poleg et al., 2019), anxiety (Masataka, 2019), insomnia (Shannon and Opila-Lehman, 2016), depression, and substance abuse (Laczkovics et al., 2021). Weight management is also a common reason for cannabinoid consumption (Bersani et al., 2016; Davis, 2016; Rossi et al., 2018). We observed a sex-specific effect on weight gain; female mice treated with CBD gained less weight over the 3-week exposure period than vehicle-treated females or male mice. Since food intake was not assessed, we cannot resolve whether these effects resulted from reduced food intake, changes in metabolic function, or a combination. However, CBD has been shown to reduce food intake in numerous rodent and human studies (Ignatowska-Jankowska et al., 2011; Farrimond et al., 2012; Devinsky et al., 2016; Linge et al., 2016). Notably, those effects were not shown to be sex specific, but stronger effects of CBD and other cannabinoids have been observed in females compared to males in both rodents and humans (Cooper and Craft, 2018; Spindle et al., 2020). Therefore, sex differences need to be accounted for in further research involving CBD and the translation to the clinic.

Our exposure period starting at PND 25 and ending at PND 45 represents the mouse brain development period similar to human adolescence (Brust et al., 2015). It’s possible that adverse effects on the developing brain and behavior could result from repeated CBD exposure earlier in development. 20 mg/kg CBD administration to pregnant dams once daily (i.e., same dose but half the exposure frequency as the current study) during gestation and lactation in rats led to heightened anxiety assessed in the marble burying task in female offspring (Wanner et al., 2021). Interestingly, the female offspring also showed improved spatial memory in the Y-maze, which is consistent with our finding of a faster spatial learning rate in the CBD-exposed cohort. Notably, we did not assess measures of cognitive flexibility or working memory that rely heavily on prefrontal cortical circuitry (Hamilton and Brigman, 2015) and undergo maturation during this adolescent time period (Klune et al., 2021), which makes them vulnerable to pharmacological perturbation.

We assessed spatial learning and memory using the Barnes Maze procedure. The hippocampus plays a critical role in spatial memory formation (Bird and Burgess, 2008) and is functionally coupled to the prefrontal cortex during navigational search strategies in the Barnes Maze (Negrón-Oyarzo et al., 2018). Hippocampal neurogenesis is important for the acquisition of spatial memory across a variety of behavioral paradigms (Lieberwirth et al., 2016), and reducing neurogenesis impairs Barnes Maze performance (Raber et al., 2004). C57BL/6J mice raised in standard laboratory housing experience sub-maximum hippocampal neurogenesis, and CBD can increase adult neurogenesis through modulating activity of cannabinoid type I receptors (CB1) (Wolf et al., 2010). This modulation may occur through inhibition of FAAH-mediated degradation of hippocampal anandamide signaling by CBD (Bisogno et al., 2001; Ibeas Bih et al., 2015). Further, this increase in neurogenesis protects against an impairment to neurogenesis and resulting anxiety resulting from a chronic stress paradigm (Campos et al., 2013). Since multiple daily i.p. injections can be stress-inducing, one potential explanation for the faster acquisition rate in the Barnes Maze by CBD-treated mice is that CBD either enhanced neurogenesis or protected against the stress-induced reduction from repeated i.p. injections. Further studies are needed to elucidate this mechanism.

CBD has over 65 known targets in the brain and body that are activated at varying doses (Ibeas Bih et al., 2015). It’s still not clear which of these targets promote CBD’s many proposed therapeutic actions, although some have been better characterized. Of particular relevance in adolescent use, some involve, though not limited to, GPR55 in epilepsies resulting from heterozygous mutation to the *Scn1a* gene (Kaplan et al., 2017), 5-HT1a receptors in anxiety (Gomes et al., 2011), depression (Linge et al., 2016), and protection against acute stressors (Resstel et al., 2009), and PPARs and TRPV receptors on digestive and gut health (Couch et al., 2019). Further, CBD inhibition of fatty acid amino hydrolase (FAAH), the primary degradation enzyme for the endocannabinoid, anandamide, is implicated or proposed to reduce compulsive behavior (Casarotto et al., 2010), anxiety (Moreira et al., 2008), borderline personality disorder (Kolla et al., 2020), and autism spectrum disorder (Aran et al., 2019). It’s reasonable to hypothesize that modulating these systems during critical stages of development could impact the brain in ways that go undetected in our broad mouse behavioral assays but may present in humans through more subtle investigative techniques or in more complex assessments. These human studies are largely lacking, but in one study of epileptic patients, subtle changes in regional white matter density were detected following 12 weeks of CBD use (Thompson et al., 2020). However, alterations were not associated with changes in cognitive function or adaptive behavioral skills. Follow-up safety studies in a human clinical population will need to be conducted in several years when currently using children and adolescents become adults.

Effective CBD dosing differs as a function of therapeutic indication. In pre-clinical acute-exposure studies, CBD reduced anxiety in the elevated plus maze at doses between 2.5 and 10 mg/kg, but this effect was lost at a higher 20 mg/kg dose in rats (Guimarães et al., 1990). We confirmed that an acute CBD dose of 5 mg/kg, but not 10 mg/kg, reduced anxiety in the elevated plus maze in mice. Our decision to test the effect of a higher, 20 mg/kg dose, was based on the presumption that a suprathreshold dose for anxiety-related behaviors, which is one of the most common reasons for off-label CBD use (Moltke and Hindocha, 2021), would impact more neural mechanisms and have more pronounced effects, should they occur. We also chose to use a suprathreshold dose for anxiety-related behavior, albeit in healthy mice, to account for dose escalation that may occur with tolerance (Long et al., 2012). This 20 mg/kg dose has been shown to improve social deficits in a mouse model expressing autism spectrum disorder behaviors (Kaplan et al., 2017). Further, clinical and pre-clinical mouse studies demonstrate CBD’s efficacy in reducing epileptic seizures at doses up to around 20 mg/kg (Devinsky et al., 2017) and 100 mg/kg (Kaplan et al., 2017), respectively. It remains possible that a higher dose than the one tested here would have more lasting effects on brain function and behavior. It is similarly possible that a lower CBD dose may contribute to more lasting effects on brain development if a high dose, by impacting different targets or modulating them at different levels, counteracted the impairing effect of a lower dose. While the evidence seems to suggest that high doses lead to more acute adverse effects than lower doses (Devinsky et al., 2018), there has been no long-term investigation of CBD’s long-term dose-response effects on brain development, thereby highlighting the need for a dose-response assessment at different developmental stages.

We report that multiple daily doses of a moderate CBD dose throughout the adolescent developmental period does not negatively impact locomotor behavior, anxiety, and spatial learning in healthy C57BL/6J mice. Further, the faster acquisition rate of a spatial learning task may highlight CBD’s potential protective benefits against stressors. While sex-specific impacts of CBD on weight gain during this period need to be considered and monitored, these findings do not detract from the progression of clinical studies investigating CBD’s therapeutic potential in an adolescent population.

## Data Availability Statement

The raw data supporting the conclusions of this article will be made available by the authors, without undue reservation.

## Ethics Statement

The animal study was reviewed and approved by Institutional Animal Care and Use Committee at Western Washington University.

## Author Contributions

JK and BP designed the experiments. JW, KR, FM, SA, MB, HS, JJ, BR, RB, TM, and EG were involved in conducting the experiments and analyzing the data. JK wrote the manuscript. All authors were involved in revising the manuscript.

## Conflict of Interest

BP is employed by Prelabs, LLC. The remaining authors declare that the research was conducted in the absence of any commercial or financial relationships that could be construed as a potential conflict of interest.

## Publisher’s Note

All claims expressed in this article are solely those of the authors and do not necessarily represent those of their affiliated organizations, or those of the publisher, the editors and the reviewers. Any product that may be evaluated in this article, or claim that may be made by its manufacturer, is not guaranteed or endorsed by the publisher.
