# Supplementary material for: Cannabidiol Exposure During the Mouse Adolescent Period Is Without Harmful Behavioral Effects on Locomotor Activity, Anxiety, and Spatial Memory
Source: Front Behav Neurosci. 2021 Aug 26;15:711639. doi: 10.3389/fnbeh.2021.711639 (PMC8426900; doi:10.3389/fnbeh.2021.711639)
Supplement: Supplementary file 1 [file Image_1.pdf]

Supplemental Figure 1

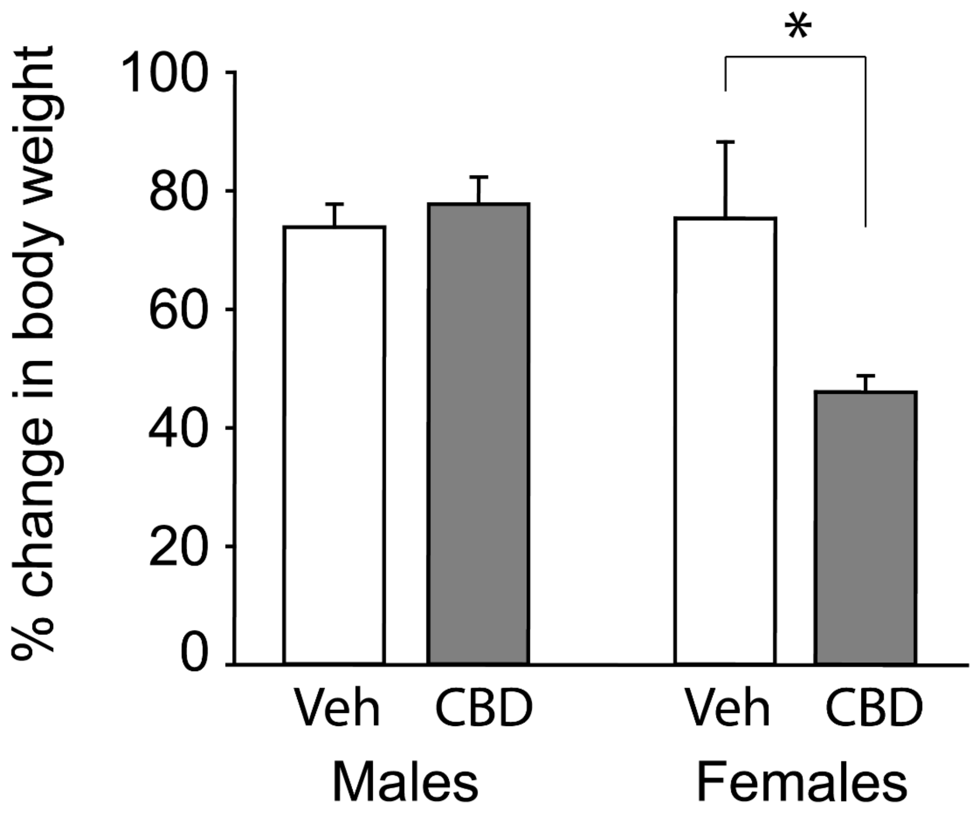

Female CBD-treated mice gained less weight during the 3-week treatment period. Summary bar chart showing the percent change in body weight from day 1 to day 21 of the treatment period in male and female mice treated with vehicle (white) or CBD (gray). Male mice treated with vehicle and CBD showed a similar percent increase in body weight (vehicle: 73.91 ± 3.87%; CBD: 77.81 ± 4.58%), whereas females treated vehicle gained a higher percent of their body weight than CBD treated mice (vehicle: 75.41 ± 12.91%; CBD: 46.14 ± 2.73%). * indicates $P < .05$ by Tukey's HSD post hoc comparisons.
