# Supplementary material for: Cannabidiol Exposure During the Mouse Adolescent Period Is Without Harmful Behavioral Effects on Locomotor Activity, Anxiety, and Spatial Memory
Source: Front Behav Neurosci. 2021 Aug 26;15:711639. doi: 10.3389/fnbeh.2021.711639 (PMC8426900; doi:10.3389/fnbeh.2021.711639)
Supplement: Supplementary file 2 [file Image_2.pdf]

Supplemental Figure 2

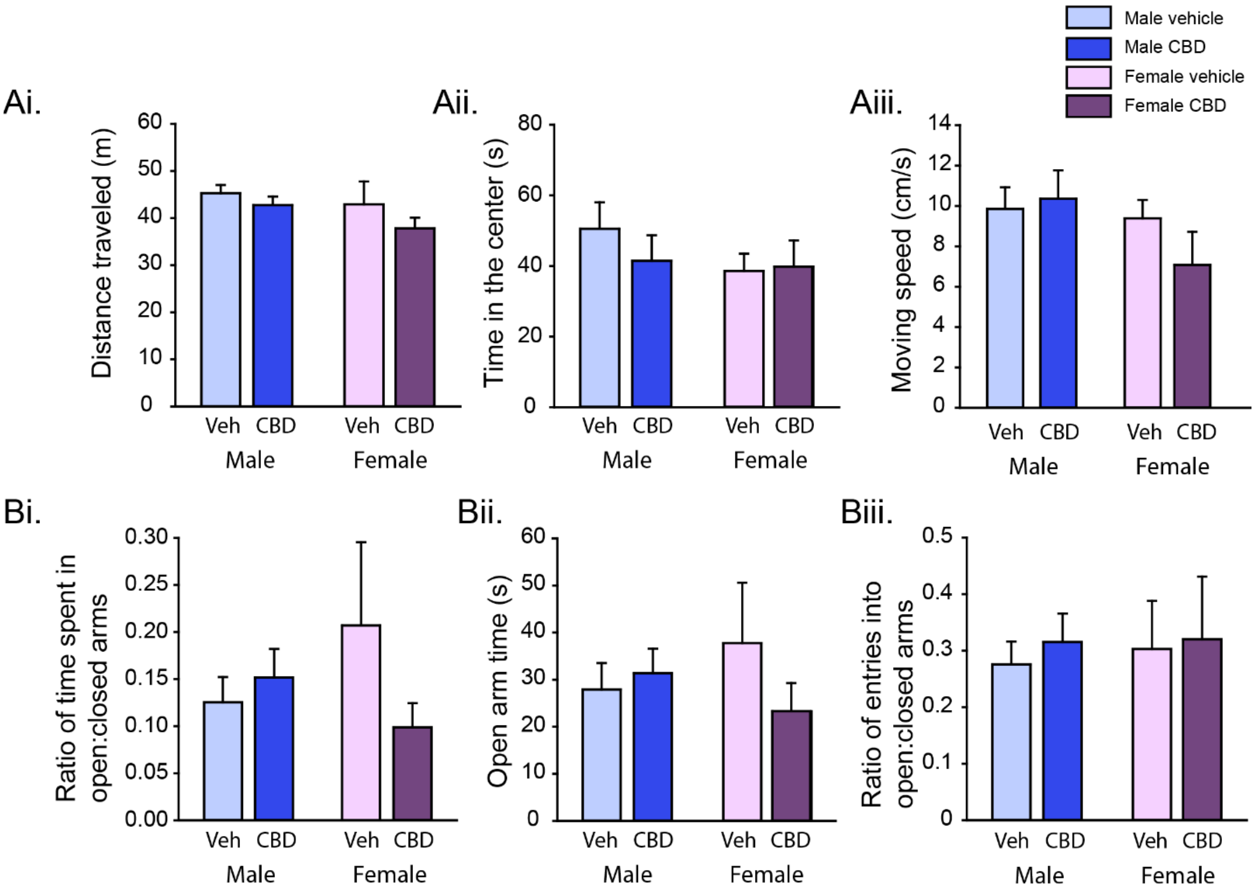

No sex differences in CBD's impact on locomotor activity in the open field or anxiety-like behavior in the EPM. For all figures, vehicle-treated males are depicted in light blue, CBD-treated males in dark blue, vehicle-treated females in light pink and CBD-treated females in dark purple. **A.** Summary bar charts of data collected in the open field indicating that there were no differences in distance traveled (Ai; males: vehicle: 45.30 ± 1.72 m; CBD: 42.72 ± 1.84 m; females: vehicle: 43.00 ± 4.82 m; CBD: 37.82 ± 2.26 m), time in the center quadrant (Aii; males: vehicle: 50.51 ± 7.49 s; CBD: 41.45 ± 7.25 s; females: vehicle: 38.54 ± 4.91 s; CBD: 39.79 ± 7.44 s), nor mean moving speed (Aiii; males: vehicle: 9.86 ± 1.07 cm/s; CBD: 9.40 ± 0.91 cm/s; females: vehicle: 10.36 ± 1.41 cm/s; CBD: 7.08 ± 1.64 cm/s). **B.** Summary bar charts of data collected in the EPM indicating that there were no differences in ratio of time spent in the open:closed arms (Bi; males: vehicle: 0.13 ± 0.03; CBD: 0.15 ± 0.03; females: vehicle: 0.21 ± 0.09; CBD: 0.10 ± 0.03), time spent in the open arms (Bii; males: vehicle: 27.93 ± 5.59 s; CBD: 31.35 ± 5.22 s; females: vehicle: 37.77 ± 12.83 s; CBD: 23.28 ± 6.00 s), nor ratio of entries into the open:closed arms (Biii; males: vehicle: 0.28 ± 0.04; CBD: 0.32 ± 0.05; females: vehicle: 0.30 ± 0.08; CBD: 0.32 ± 0.11). Data are represented as mean ± SEM.
