# Supplementary material for: Cannabidiol Exposure During the Mouse Adolescent Period Is Without Harmful Behavioral Effects on Locomotor Activity, Anxiety, and Spatial Memory
Source: Front Behav Neurosci. 2021 Aug 26;15:711639. doi: 10.3389/fnbeh.2021.711639 (PMC8426900; doi:10.3389/fnbeh.2021.711639)
Supplement: Supplementary file 3 [file Image_3.pdf]

Supplemental Figure 3

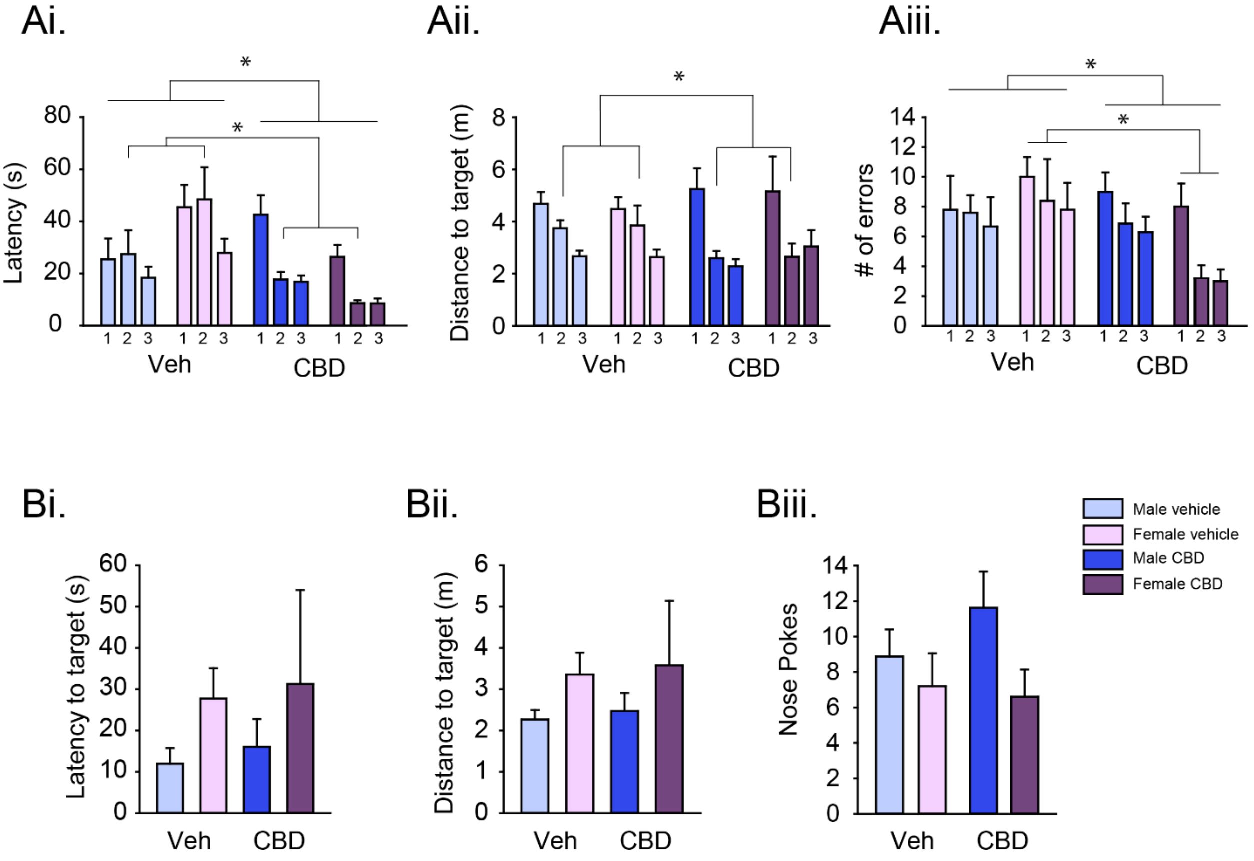

Barnes Maze performance organized by sex. For all figures, vehicle-treated males are depicted in light blue, CBD-treated males in dark blue, vehicle-treated females in light pink and CBD-treated females in dark purple. **A.** Summary bar charts showing the mean latency to the escape box (Ai), mean distance to the escape box (Aii), and mean number of errors (Aiii) for each day of the acquisition period. See Table 1 for descriptive statistics. **B.** Summary bar charts showing the latency (Bi; male: vehicle: 11.97 ± 3.79 s; CBD: 16.08 ± 6.69 s; female: vehicle: 27.74 ± 7.35 s; CBD: 31.30 ± 6.69 s), distance to the target hole (Bii; male: vehicle: 2.27 ± 0.23 m; CBD: 2.47 ± .44 m; female: vehicle: 3.36 ± .53 m; CBD: 3.58 ± 1.56 m), and number of nose pokes into the target hole (Biii; male: vehicle: 8.88 ± 1.53; CBD: 11.63 ± 2.04; female: vehicle: 7.20 ± 1.86; CBD: 6.60 ± 1.54) during the probe day. Date are represented as the mean ± SEM. * indicates $P < .05$ by Tukey's HSD post hoc comparisons.
